# Thiamin supplementation does not reduce the frequency of adverse events after anti-malarial therapy among patients with falciparum malaria in southern Laos

**DOI:** 10.1186/1475-2875-13-275

**Published:** 2014-07-15

**Authors:** Mayfong Mayxay, Maniphone Khanthavong, Lorna Cox, Odai Sichanthongthip, Mallika Imwong, Tiengkham Pongvongsa, Bouasy Hongvanthong, Samlane Phompida, Viengxay Vanisaveth, Nicholas J White, Paul N Newton

**Affiliations:** 1 Lao-Oxford-Mahosot Hospital-Wellcome Trust Research Unit (LOMWRU), Microbiology Laboratory, Mahosot Hospital, Vientiane, Lao People’s Democratic Republic; 2 Faculty of Postgraduate Studies, University of Health Sciences, Vientiane, Lao People’s Democratic Republic; 3 Centre for Clinical Tropical Medicine, Churchill Hospital, University of Oxford, Oxford, UK; 4 Centre of Malariology, Parasitology and Entomology, Vientiane, Lao People’s Democratic Republic; 5 MRC Human Nutrition Research, Cambridge, UK; 6 Department of Molecular Tropical Medicine and Genetics, Faculty of Tropical Medicine, Mahidol University, Bangkok, Thailand; 7 Savannakhet Provincial Malaria Station, Savannakhet Province, Lao People’s Democratic Republic; 8 Faculty of Tropical Medicine, Mahidol University, Bangkok, Thailand

**Keywords:** *Plasmodium falciparum*, Malaria, Treatment, Thiamin, Laos

## Abstract

**Background:**

In a recent study one third of Lao patients presenting with uncomplicated *Plasmodium falciparum* malaria had biochemical evidence of thiamin deficiency, which was associated with a higher incidence of adverse events. Thiamin supplementation might, therefore, reduce adverse events in this population.

**Methods:**

An exploratory, double-blind, parallel group, placebo-controlled, superiority trial of thiamin supplementation in patients of all ages with uncomplicated and severe falciparum malaria was conducted in Xepon District, Savannakhet Province, southern Laos. Patients were randomly assigned to either oral thiamin 10 mg/day for 7 days immediately after standard anti-malarial treatment then 5 mg daily until day 42, or identical oral placebo.

**Results:**

After interim analyses when 630 patients (314 in thiamin and 316 in placebo groups) had been recruited, the trial was discontinued on the grounds of futility. On admission biochemical thiamin deficiency (alpha ≥ 25%) was present in 27% of patients and 9% had severe deficiency (alpha > 31%). After 42 days of treatment, the frequency of thiamin deficiency was lower in the thiamin (2%, 1% severe) compared to the placebo (11%, 3% severe) groups (p < 0.001 and p = 0.05), respectively. Except for diarrhoea, 7% in the placebo compared to 3% in the thiamin group (p = 0.04), and dizziness on day 1 (33% vs 25%, p = 0.045), all adverse events were not significantly different between the groups (p > 0.05). Clinical, haematological, and parasitological responses to treatment did not differ significantly between the two groups.

**Conclusion:**

Thiamin supplementation reduced biochemical thiamin deficiency among Lao malaria patients following anti-malarial drug treatment, but it did not reduce the frequency of adverse events after anti-malarial therapy or have any detected clinical or parasitological impact.

**Trial registration:**

ISRCTN 85411059

## Background

Malaria remains an important cause of morbidity and mortality in rural Lao PDR (Laos) particularly in the southern provinces [1-3]. In 2005, the Lao government changed the nationally recommended treatments for uncomplicated *Plasmodium falciparum* malaria from chloroquine (CQ) and sulphadoxine-pyrimethamine (SP) to artemisinin-based combination therapy (ACT) (artemether-lumefantrine), which has been highly efficacious [2,3].

Malaria morbidity and mortality burden is increased by micronutrient deficiencies and malnutrition [4]. In western Thailand, adults with malaria, especially those with severe malaria, had biochemical evidence for thiamin deficiency [5]. One third of Lao patients presenting with uncomplicated falciparum malaria in southern Laos had evidence for biochemical thiamin deficiency and 12% had unequivocal evidence for severe deficiency [6]. Infantile beriberi is common in some communities in Laos [7,8] but there are no data on biochemical thiamin deficiency in older Lao children or adults without malaria. In the malaria studies the proportion of patients with biochemical thiamin deficiency was significantly lower at 42 day follow up, but patients received thiamin supplementation during recovery so it was uncertain whether this reduction was a consequence of recovery from malaria or thiamin supplementation or both. In a post-hoc analysis (unpublished), the estimated median (range) admission basal erythroycte transketolase activity (ETK) but not the activation coefficient (alpha), was significantly lower in those who developed one or more adverse effects during or after treatment than those who did not (p = 0.003). This measure was significantly lower in those with dizziness, headache, and nightmares than in those without (p < 0.001). Shortness of breath before treatment was associated with lower median (range) admission extrapolated basal ETK (p = 0.002), whilst shortness of breath after treatment was not. In the Wenicke-Korsakoff syndrome, ataxia is a cardinal feature and dizziness has been reported [9]. Thiamin, especially as thiamin triphosphate, is important in brain function [10]. Nightmares have not been described previously in beriberi or subclinical thiamin deficiency. Whether the associations of adverse events with subclinical thiamin deficiency are related to the drug or to malaria or interaction between the two factors is unclear. That dizziness was associated with low extrapolated basal ETK both before and after anti-malarial treatment suggests that the association may be a consequence of malaria and not the anti-malarial treatment. In the earlier studies in Laos Mayxay *et al.*[6] did not include patients with severe malaria, young children or pregnant women, who would be expected to have a higher incidence of thiamin deficiency. Neurological adverse events, whether attributable to malaria and anti-malarial drugs, are important aspects of morbidity during malaria and recovery [11] and any measure to reduce their frequency would be of public health importance.

An exploratory, double-blind, parallel group, placebo-controlled, superiority trial of thiamin supplementation was conducted in patients of all ages with uncomplicated and severe falciparum malaria to assess the frequency and severity of biochemical thiamin deficiency and its relationship to malaria severity and whether thiamin supplementation reduces the frequency of adverse events after anti-malarial therapy.

## Methods

### Trial design, patients, clinical and laboratory procedures

This was an exploratory, double-blind, parallel group, placebo-controlled, randomized (variable blocks), superiority trial conducted between June and November from 2008–2010 at Xepon (30 beds) Inter-District Hospital (16.69° N, 106.20° E, 208 metres above mean sea level), Savannakhet Province, ~665 km south-east of Vientiane, the capital of Laos. Xepon (88 villages, population 48,000) is inhabited predominantly by rice farmers of the Lao Theung ethnic groups. Malaria transmission is seasonal with a peak during the rainy months of July and August [2,3].

In previous prospective trials at Phalanxay Clinic in the same Province (~60 Km west of Xepon), 247/550 (49%) patients had at least one potential adverse event noted [12,13]. A sample size calculation assuming that 50% of patients would have a potential adverse event, aiming for a power of 80% and level of significance of 5% and a clinically significant difference of 20% in adverse effect frequency, with a ~5% drop out rate, suggested that 814 patients should be recruited (407 to each arm). A follow up of 42 days was planned.

Patients of any age were included in the trial provided that they or their guardians (in the case of children) gave informed written consent, had microscopically-confirmed *P. falciparum* infection or mixed *Plasmodium* species infections, with a history of fever, were willing and expected to be able to comply with the study protocol for the duration of the 42 days follow up, and had not taken a full course of any anti-malarial drugs in the previous three days. Patients were excluded if they had a history of hypersensitivity to thiamin or artemether-lumefantrine, presented with intercurrent non-malarial illness, had clinically apparent suspected thiamin deficiency (beriberi) [14] or any condition, which in the judgment of the investigator would place the subject at undue risk or interfere with the results of the study.

Patients’ admission clinical details including current and past medical history, pre-treatment, and findings from physical examination were recorded on the case record form. Venous blood samples were taken for the base line assay of washed red cell ETK (see below), parasite counts, haematocrit, blood glucose, and lactate, and three blood spots were collected on 3MM filter paper (Whatman, Maidstone, UK) and stored in a plastic bag with silica gel for later malaria genotype analysis [2,15]. For women of child-bearing age a urinary pregnancy test (Orchid+, True Line Med Co., Ltd, Bangkok, Thailand) was performed before anti-malarial treatment was prescribed. Children were defined as those aged <15 years.

Randomization was 1:1 to either thiamin or identical placebo. The random numbers, generated by a study doctor who did not enrol patients, corresponded to an opaque sealed envelope and plastic tablet container containing either thiamin tablets or visually identical placebos, prepared by a pharmacist not involved in patient recruitment. Only the study doctor who performed the randomization held the master randomization list and knew the contents of each container. If the study criteria were met, patients were admitted to the hospital and randomized (in variable blocks – the assigned treatment number in a sealed opaque envelope was opened only after the decision to recruit had been made and consent form signed) to receive study drugs: either oral thiamin (5 mg capsule) (Olan-Kemed Co.Ltd, Bangkok, Thailand) two capsules immediately after anti-malarial drugs, followed by two capsules daily for seven days followed by one capsule daily until day 42 or identical placebo, which had the same appearance as the thiamin capsule and was manufactured by the same company (Olan-Kemed Co. Ltd, Bangkok, Thailand).

Ingestion of both anti-malarials and study drugs was taken within < 20 minutes after enrolment and was directly observed for patients in hospital and capsule containers checked for the number of capsules left at each subsequent visit. Patients with severe malaria who were unable to take oral medication started oral thiamin or placebo (according to the randomization above) as soon as they were able to. Severe malaria was defined according to the World Health Organization [16].

Malaria was treated with the nationally recommended anti-malarials [17]. Patients with uncomplicated disease and pregnant patients in the 2^nd^ or 3^rd^ trimesters who were able to swallow tablets, were treated with artemether (20 mg)-lumefantrine (120 mg) (AL, Co-artem®, Novartis): one dose twice daily for three days. Dosing by body weight were one tablet if <15 kg, two tablets if 15–24 kg, three tablets if 25–34 kg, and four tablets if ≥35 kg. All patients were asked to take fatty food with the anti-malarial drugs and drug administration was directly observed by a study nurse. For children who could not swallow tablets, the appropriate drug dose was crushed and mixed with water and given in a syringe. Severe malaria patients were treated with artesunate (Guilin Pharmaceutical Co. Ltd.) 2.4 mg/kg IV stat, followed by 2.4 mg/kg IV at 12 hours and 24 hours and then daily until able to take oral medication when they were given artemether-lumefantrine for 3 days as above. Pregnant patients in the first trimester were given quinine (Thai Government Pharmaceutical Organization [GPO]) plus clindamycin (Thai GPO) (WHO 2006). Patients who had recurrent *P. falciparum* parasitaemia and did not have contraindications to mefloquine were treated with artesunate 4 mg/kg/day in single daily dose for 3 days (D0-D2) (Guilin Pharmaceutical Co. Ltd.) + mefloquine 15 mg/kg on D1 and 10 mg/kg on D2 in single daily doses (Lariam™, Roche Co.). Patients with contraindications to mefloquine and were aged >8 years, were treated with artesunate (as above) and doxycycline (Bangkok Lab & Cosmetic Co., Ltd, Bangkok, Thailand) 4 mg/kg/day for 7 days. Those who had *P. vivax* appearance during follow up were treated with chloroquine (Thai GPO) (25 mg base/kg) over three days and further followed up.

Patients were observed for at least one hour after drug administration and all episodes of vomiting recorded. If vomiting occurred within 30 minutes of anti-malarial and/or thiamin/placebo administration, the full dose was repeated. Patient who vomited within 30 minutes to one hour, a half dose was re-administered. If the patient vomited >2 times within one hour, he/she was treated as for severe malaria (see above).

Vital signs (axillary temperature, pulse, respiratory rate, and blood pressure) were measured every six hours. Patients and parasite counts were reviewed daily until two consecutive negative blood smears, then weekly for 42 days from the start of treatment or at other times if patients felt unwell. Patients were discharged only when their fever and parasitaemia had cleared (defined as axillary temperature <37.5°C and 0 parasite per 500 white cells on thick film after two consecutive negative slides, respectively) and were asked to return to the hospital at days 7, 14, 21, 28, 35 and 42 (or at any time the patients feel unwell) or were visited at home in case the patients did not return as planned.

At each weekly visit, the symptoms of the patients during the previous week were recorded. Details of any new medications taken were recorded and medicines examined to check whether they contained thiamin. Finger-prick blood samples were taken for malaria blood smears and haematocrit and blood spots were collected onto filter paper strips from all patients with recurrent fever or malaria symptoms. One ml of heparinized venous blood samples was taken from all patients at day 42 for convalescent ETK. PCR amplification was performed on paired samples for parasite genotyping to distinguish between reinfection and recrudescence using three parasite loci (MSP-1, MSP-2, and GLURP) [15]. The PCR tests were performed at the Mahidol-Oxford Tropical Medicine Research Unit (MORU) malaria molecular laboratory, Bangkok, Thailand. Fingerprick blood glucose and lactate were determined using Accu-Chek Advantage II (Roche Diagnostics Manufactures) and Accutrend® (Roche Diagnostics Manufactures) devices, respectively.

The occurrence of adverse events was determined prospectively. Anorexia, nausea, headache, vomiting, dreams, nightmares, abdominal pain, paraesthesia, ability to rise from squatting position, diarrhoea, vertigo, rash and itch were asked about at each visit. A visual analogue scale (VAS; 0–10) was used to measure the severity of headache and vertigo. Particular attention was given to record and distinguish dreams and nightmares (unpleasant dreams as defined by the patient) and these were only asked from those aged >4 years. A cardiovascular examination, looking for signs of heart failure, was performed on admission and days 2, 7 and 42. A brief neurological examination was performed on admission, on day 2 and then on days 7 and 42. This included tests for coordination (heel-toe ataxia), fine finger dexterity (ability to pick up a 500 mg paracetamol tablet that is 10 mm diameter and 0.5 mm thick), nystagmus and balance (assessment of sway when standing with feet together and eyes shut) [18] and ability of those >5 years old to rise from a squatting position.

### Red cell transketolase activity assays

Immediately after collection, the lithium heparin anticoagulated blood was centrifuged and washed in phosphate buffered saline three times, with removal of the buffy coat initially and after each wash. Washed red cell samples were stored at -30°C for maximum of 3 months and then at -70°C until shipment to the UK on dry ice. Red cell transketolase activity assays were performed in Cambridge using an adaptation of the method of Vuilleumier *et al*. [19] as described in Khounnorath *et al*. [7] and Soukaloun *et al*. [8]. These are the functional assays for the thiamin-dependent erythrocyte transketolase enzyme in washed red cells. The activation coefficient (alpha) is the ratio of *in vitro* erythrocyte transketolase activity (ETK) after thiamin pyrophosphate has been added minus the basal ETK before thiamin pyrophosphate has been added, to the basal ETK, expressed as a percentage. Higher alpha coefficients represent greater degrees of thiamin deficiency. In contrast to an earlier study Mayxay *et al*. [6] haemoglobin was measured to calculate ETK [19]. Categories of α of <15, 15–24, 25–31 and >31% were used with α >31% [5,6] defining severe biochemical deficiency. Although previous studies in Laos [7,8] demonstrated that basal ETK and to a lesser extent activated ETK, and not the alpha coefficient, is the best biochemical marker of thiamin deficiency in infants, the alpha value was used to determine whether patients were thiamin deficient because the majority (97%) of recruited patients were not infants.

Written informed consent was obtained from all participants. Ethical clearance for the study was granted by the Lao National Ethics Committee for Health Research and the Oxford University Tropical Medicine Research Ethics Committee (OXTREC). The trial is registered as ISRCTN: 85411059. A Data and Safety Monitoring committee (DMC), including a statistician familiar with clinical trials and three doctors familiar with thiamin deficiency, malaria and clinical trials, was set up to perform interim analyses at the end of each trial season (the rainy season). The trial was monitored by the Clinical Trials Support Group of MORU, Bangkok, Thailand.

### Outcome measures

The primary objective of the trial was to determine whether the frequency of adverse events, after initiation of anti-malarial treatment, was significantly lower in those who received thiamin supplementation in comparison to those who did not. The secondary objective was to determine the frequency of biochemical thiamin deficiency and whether this was related to the clinical severity of disease and to compare resolution of deficiency between those who did and did not receive thiamin supplementation. The following outcomes were also reported: PCR-corrected adequate clinical and parasitological responses at day 42 [20], the PCT (the time in hours from the first treatment dose to the first of two consecutive thick films that were negative for asexual falciparum parasites after checking ≥500 oil fields) and fever clearance times (FCT, time in hours from the start of treatment at which the tympanic temperature first dropped below 37.5°C and remained below 37.5°C for 48 hours) following anti-malarial treatment. These have been reported, in part, in Mayxay *et al.*[3].

### Statistical analysis

Data were analysed using Stata v9 (StataCorp, College Station, TX, USA). Comparisons between two groups were made by the Mann–Whitney *U*, Student’s *t*, chi-square, and Fisher’s exact tests, as appropriate. Cure rates were calculated as the proportion of patients with PCR-confirmed recrudescence using intention-to-treat (ITT) and per-protocol (PP) populations and by Kaplan-Meier survival analysis. In the ITT population all losses to follow-up were treated as failures and in the PP population losses to follow-up were excluded from the analysis. Patients with new infections were regarded as cures in both analyses. Gametocyte carriage was summarized as person-gametocytes-week rates calculated as the total number of weeks with gametocytes divided by the total number of weeks of follow-up.

## Results

**Figure 1:**
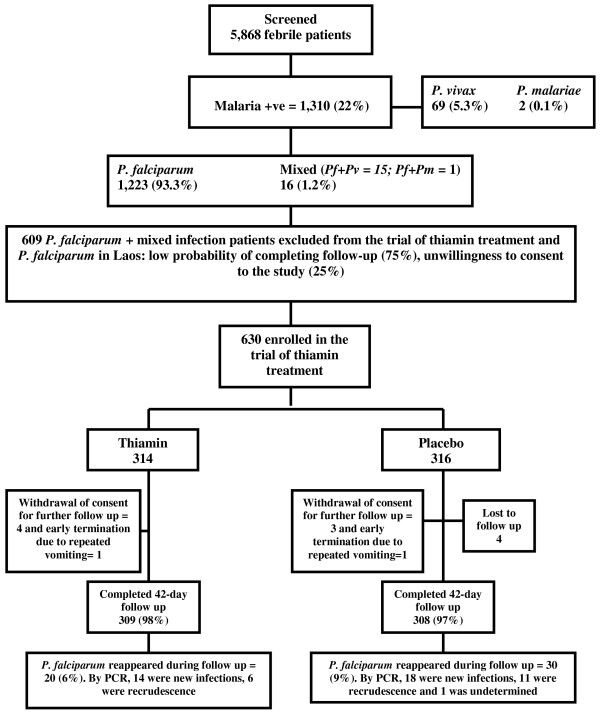
**Patient flow diagram.** N.B. +ve = positive; Pf = *Plasmodium falciparum*; Pv = *P. vivax*; Pm = *P. malariae*; PCR = polymerase chain reaction.

At the end of the third trial season when 630 patients had been recruited, following an interim analysis, the DMC advised that the study should be discontinued on the grounds of futility, with evidence that the study could not meet its primary endpoint. Of 630 patients enrolled in the trial [619 (98%) with *P. falciparum* mono-infection and 11 (2%) with mixed infections (10 *P. falciparum* + *Plasmodium vivax* and one *P. falciparum* + *Plasmodium malariae*)], 314 and 316 received thiamin and identical placebo, respectively (Figure 1). Forty-two day follow up was completed for 309 (98%) in the thiamin and 308 (97%) in the placebo groups (Figure 1). Two patients (one in each arm) had persistent vomiting after taking AL, 7 (4 in thiamin and 3 in placebo groups) withdrew consent for further follow up, and 4 in the placebo group were lost to follow up.

### Analysis of thiamin intervention medicine and patient adherence

High-performance liquid chromatography (HPLC), using the method of the United States Pharmacopeia [21], of three randomly selected thiamin tablets gave %API of 91, 93, and 92%. Capsule counts at visits following discharge from hospital suggested that 6% of all patients (5% in thiamin and 6% in placebo groups, p = 0.62) did not finish their thiamin/placebo capsules. The patients stated that the reason for not finishing them was that they forgot. The overall median (range) days that the patients forgot to take the capsules were 3 (1–8) and this was not different between thiamin [3 (1–7) days] and placebo [3.5 (1–8) days] groups (p = 0.94).

### Demographic, clinical and laboratory details on admission

Patient admission demographic, clinical and laboratory details differed little between the thiamin and placebo groups (Table 1). All patients in both groups had a history of fever before admission and 471 (75%) were febrile at the time of enrollment. Twenty-nine (5%) patients were pregnant, 414 (66%) were children, 21 (3%) were infants, and 34 (5%) had severe malaria [16]. The admission symptoms and signs, and median (range) headache and dizziness visual analogue scores were not different between two groups (Table 2).

**Table 1:** **Admission demographic, clinical and laboratory details for patients included in the study on thiamin treatment and *Plasmodium falciparum *** **malaria in Laos**

**Variables**	**Treatment groups**		
	**All (N = 630)**	**Thiamin (n = 314)**	**Placebo (n = 316)**
Sex, M, no (%)	317 (50)	155 (49)	162 (51)
Age, years, median (range)	8 (0.5 – 73)	9 (0.5 – 63)	8 (0.7 – 73)
No. (%) patients aged <15 years	414 (66)	204 (65)	210 (66)
Body weight, kg	28.1 (26.8 – 29.4)	28.7 (26.8 – 30.7)	27.5 (25.6 – 29.3)
Height, cm	122.1 (119.9 – 124.4)	122.8 (119.5 – 126.0)	121.5 (118.5 – 124.8)
Previous malaria attack, no. (%) of patients^a^	166 (26)	85 (27)	81 (26)
Axillary temperature, °C	38.4 (38.3 – 38.5)	38.4 (38.3 – 38.5)	38.5 (38.3 – 38.6)
Patients without fever on admission, no. (%)	159 (25)	85 (27)	74 (23)
Systolic blood pressure, mm Hg	100.8 (99.8 – 101.9)	100.7 (99.2 – 102.2)	100.9 (99.4 – 102.4)
Diastolic blood pressure, mm Hg	68.8 (68.0 – 69.7)	68.9 (67.6 – 70.1)	68.8 (67.7 – 70.0)
Pulse, beats/min	96.4 (95.2 – 97.5)	96.0 (94.4 – 97.6)	96.7 (95.2 – 98.3)
Respiratory rate/min	28.1 (27.6 – 28.6)	27.8 (27.1 – 28.6)	28.4 (27.7 – 29.1)
Glasgow coma score, median (range)	15 (4 – 15)	15 (12 – 15)	15 (4 – 15)
Severe malaria, no. (%) [WHO, 2000]	34 (5)	18 (6)	16 (5)
Pregnancy, no. (%)	29 (5)	13 (4)	16 (5)
Headache visual analogue score*	5.2 (5.0 – 5.5)	5.3 (5.0 – 5.7)	5.1 (4.7 – 5.7)
Dizziness visual analogue score, median (range)*	4 (0 – 10)	4 (0 – 10)	4 (0 – 10)
Splenomegaly, no. (%) of patients	163/628 (26)	85/313 (27)	78/315 (25)
Hepatomegaly, no (%) of patients	143/627 (23)	63/313 (20)	80/314 (25)
Lymphadenopathy, no (%) of patients	2/629 (0.3%)	1/313 (0.3%)	1/316 (0.3)
Parasitaemia, geometric mean parasites/μL (95%CI)	25,677 (22,350 – 29,499)	27,775 (22,995 – 33,549)	23,750 (19,369 – 29,123)
Mixed malaria species infection, no. (%)	11 (2)	5 (2)	6 (2)
Gametocytaemia, no. (%) of patients	27 (4)	14 (4)	13 (4)
Gametocytaemia, geometric mean parasite/μL (95%CI)	255 (125 – 521)	198 (78 – 503)	334 (98 – 1,134)
Haematocrit, %	35.6 (35.0 – 36.1)	35.6 (34.8 – 36.4)	35.5 (34.7 – 36.3)
Glucose, mmol/L	6.1 (6.0 – 6.2)	6.2 (6.1 – 6.3)	6.0 (5.9 – 6.2)
Lactate, mmol/L	3.2 (3.1 – 3.3)	3.1 (3.0 – 3.2)	3.3 (3.1 – 3.4)

NOTE. Data are presented as mean values (95%CI), unless otherwise indicated. ^a^Defined as patient or patient’s guardian reporting that the patient had had a febrile illness with a positive malaria slide. *Only children aged ≥ 4 years were asked about these symptoms.

**Table 2:** **Admission symptoms and signs for patients included in the study on thiamin treatment and *Plasmodium falciparum *** **malaria in Laos**

**Symptoms and signs**	**All (N = 630)**	**Treatment groups**		
		**Thiamin (n = 314)**	**Placebo (n = 316)**	***P*-value**
Headache*	403/439 (92)	203/218 (93)	200/221 (90.5)	0.32
Headache visual analogue	5 (0 – 10)	5 (5 – 10)	5 (0 – 10)	0.60
score: median (range)*				
Chill	569/630 (90)	281/314 (89)	288/316 (91)	0.48
Myalgia*	246/438 (56)	127/218 (58)	119/220 (54)	0.38
Weakness	600/629 (95)	297/313 (95)	303/316 (96)	0.55
Dizziness*	324/438 (74)	162/218 (74)	162/220 (74)	0.87
Dizziness visual analogue	4 (0 – 10)	4 (0 – 10)	4 (0 – 10)	0.98
score: median (range)*				
Vertigo*	124/438 (28)	62/218 (28)	62/220 (28)	0.95
Tinnitus*	93/438 (21)	54/218 (25)	39/220 (18)	0.07
Anorexia	567/630 (90)	284/314 (90)	283/316 (90)	0.71
Nausea*	295/440 (67)	146/219 (67)	149/221 (67)	0.87
Vomiting	343/630 (54)	171/314 (54)	172/316 (54)	0.99
Abdominal pain*	123/448 (27)	64/220 (29)	59/228 (26)	0.44
Diarrhoea	130/629 (21)	74/314 (24)	56/315 (18)	0.07
Insomnia*	372/630 (59)	185/314 (59)	187/316 (59)	0.98
Nightmare*	40/438 (9)	19/218 (9)	21/220 (10)	0.76
Palpitation*	186/439 (42)	95/219 (43)	91/220 (41)	0.67
Dyspnoea*	160/627 (25.5)	71/312 (23)	89/315 (28)	0.11
Cough	142/630 (22)	60/314 (19)	82/316 (26)	0.04
Sore throat*	55/447 (12)	24/222 (11)	31/225 (14)	0.34
Irritable	22/628 (3.5)	9/313 (3)	13/315 (4)	0.39
Rash	1/630 (0.2)	1/314 (0.3)	0	0.31
Urticaria	4/630 (0.6)	2/314 (0.6)	2/316 (0.6)	0.99
Itch	4/630 (0.6)	1/314 (0.3)	3/316 (1)	0.32
Chest abnormality**	2/630 (0.3)	0	2/316 (0.6)	0.16
Drowsiness	19/630 (3)	10/314 (3)	9/316 (3)	0.80
Seizure	21/630 (3)	12/314 (4)	9/316 (3)	0.49
Jaundice	1/630 (0.2)	1/314 (0.3)	0	0.31

NOTE. Data are shown as number (%) unless otherwise indicated, *Only children aged ≥ 4 years were asked about these symptoms, **Chest abnormality: crepitation, rhonchi.

### Outcome measures

Fever and parasite clearance times, post-treatment haematocrit at all time points and gametocyte rates were not significantly different between patients who received thiamin and placebo (Table 3). Baseline (day 0) and day 35 and 42 mean haematocrit improved significantly after treatment for both groups (Paired *t*-test p < 0.001 for both groups, Table 3).

**Table 3:** **Outcome measures for the treatment of patients included in the study on thiamin treatment and *Plasmodium falciparum *** **malaria in Laos†**

**Variables**	**Treatment groups**		
	**All (N = 630)**	**Thiamin (n = 314)**	**Placebo (n = 316)**
*P. falciparum* recurrence, no. (%)^a^	50 (8)	20 (6)	30 (9)
42-day cure rate, no. (%) of patients^a^	612/630 (97)	308/314 (98)	304/316 (96)
42-day cure rate per protocol, no. (%) of patientsΨ	599/617 (97)	303/309 (98)	296/308 (96)
Fever clearance time, mean hours (95%CI)^a,b^	26.5 (25.1 – 27.9)	26.6 (24.7 – 28.6)	26.4 (24.4 – 28.4)
Patients remained febrile at day 1, no. (%)	317 (50)	157 (50)	160 (51)
Patients remained febrile at day 2, no. (%)	86 (14)	44 (14)	42 (13)
Parasite clearance time, median days (range)^a,c^	1 (1–3)	1 (1–3)	1 (1–3)
Positive parasitaemia at day 1, no. (%) of patients	191/627 (30)	98/311 (31.5)	93/316 (29)
Positive parasitaemia at day 2, no. (%) of patients	1	0	1
Gametocytaemia detected at anytime, no. (%) of patients	31 (5)	15 (5)	16 (5)
Gametocytaemia after treatment, no. (%) of patients	4 (0.6)	1 (0.3)	3 (1)
Gametocyte clearance time, median days (range)^d^	7 (1 – 21)	7 (1–14)	7 (7 – 21)
Gametocyte cleared by day 7, no. (%)^d^	28 (90)	14 (93)	14 (87.5)
Median (range) gametocyte-person-weeks^d^	0.42 (0.14 – 1.0)	0.42 (0.14 – 1.0)	0.42 (0.14 – 0.42)
*P. vivax* appearance after treatment for *P. falciparum*, no. (%) of patients	21/630 (3)	8/314 (2.5)	13/316 (4)
Day 0 haematocrit, mean % (95%CI)	35.6 (35.0 – 36.1)	35.6 (34.8 – 36.4)	35.5 (34.7 – 36.3)
Day 1 haematocrit, mean % (95%CI)	32.0 (31.5 – 32.5)	32.1 (31.4 – 32.8)	31.9 (31.1 – 32.6)
Day 2 haematocrit, mean % (95%CI)	30.7 (30.2 – 31.2)	30.9 (30.2 – 31.6)	30.6 (29.9 – 31.3)
Day 3 haematocrit, mean % (95%CI)	30.7 (30.2 – 31.2)	30.9 (30.2 – 31.6)	30.6 (29.9 – 31.3)
Day 7 haematocrit, mean % (95%CI)	32.9 (32.5 – 33.3)	33.1 (32.5 – 33.7)	32.7 (32.1 – 33.3)
Day 14 haematocrit, mean % (95%CI)	34.0 (33.7 – 34.4)	34.1 (33.6 – 34.7)	33.9 (33.4 – 34.5)
Day 21 haematocrit, mean % (95%CI)	35.4 (35.0 – 35.0)	35.4 (34.9 – 35.9)	35.3 (34.8 – 35.8)
Day 28 haematocrit, mean % (95%CI)	35.9 (35.6 – 36.3)	36.0 (35.5 – 36.5)	35.8 (35.4 – 36.3)
Day 35 haematocrit, mean % (95%CI)	36.3 (36.0 – 36.7)	36.3 (35.8 – 36.8)	36.3 (35.8 – 36.8)
Day 42 haematocrit, mean % (95%CI)	37.5 (37.1 – 37.8)	37.5 (37.0 – 38.0)	37.5 (37.0 – 38.0)

NOTE. †No statistical difference was found between thiamin and placebo groups.

ΨThe patient with undetermined PCR result was classified as recrudescent infection.

^a^Intention-to-treat analysis.

^b^Data were available from 295 and 306 patients in thiamin and placebo groups, respectively.

^c^Data were available from 311 and 316 patients in thiamin and placebo groups, respectively.

^d^Data were available from 15 and 16 patients in thiamin and placebo groups, respectively.

Of 50 (8%) patients (20 in thiamin and 30 in placebo arms) with subsequent *P. falciparum* reappearance, PCR genotyping indicated that 17/50 (34%) had recrudescent infections [6/20 (30%) in thiamin and 11/30 (37%) in placebo groups, (p = 0.64)]. One patient in placebo group with *P. falciparum* reappearance had an indeterminate PCR result and, considering this patient as having a recrudescent infection, the overall 42-day cure rates per protocol, excluding patients who were lost to follow up, withdrew consent, had persistent vomiting, or re-infection, were 97% (599/617) [98% (303/309) for thiamin and 96% (296/308) for placebo groups, p = 0.16] [3].

Including the patients with repeated vomiting, those lost to follow up or withdrew (censored at the time last seen), the undetermined PCR result, and recrudescences all as failures gave 42-day PCR uncorrected cure rates (95%CI) by survival analysis (Kaplan-Meier) of 93.5% (90.1 – 95.8%) for thiamin and 90.3% (86.5 – 93.1%) for placebo groups. For the PCR adjusted survival analysis, the cure rates (95%CI) were 98.0% (95.7 – 99.1%) and 96.1% (93.2 – 97.8%) for thiamin and placebo groups, respectively [3].

By conventional ITT analysis, the overall 42-day cure rates, adjusted for re-infection, were 97% (612/630) [98% (308/314) for thiamin and 96% (304/316) for placebo groups, p = 0.15]. Of 18 patients (6 in thiamin and 12 in placebo groups) considered as having recrudescent infection, 16 (6 in thiamin and 10 in placebo groups) had parasite recurrence at or before day 28. The 28-day cure rates by ITT analysis were 308/314 (98%) for thiamin and 306/316 (97%) for placebo groups. The median (range) interval to recrudescent falciparum infections was 21 (14–42) days. By ITT and per protocol analyses, the 42-day PCR uncorrected cure rates for thiamin and placebo groups were 294/314 (94%) *vs* 286/316 (90.5%); (p = 0.15), and 289/309 (94%) *vs* 278/308 (90%), (p = 0.14), respectively. There were 21 episodes of vivax malaria during the follow-up period, of which 8 (38%) were in the thiamin group and the median (range) interval to *P. vivax* appearance was 30 (21–42) days [3].

### Thiamin status before and after anti-malarial and thiamin/placebo treatments

**Figure 2:**
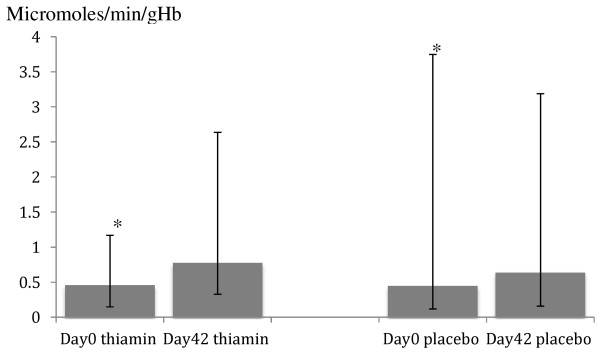
**Basal erythrocyte transketolase activity in micromoles/min/gHb, shown as median in histogram and range in error bar.** (*significant difference from D42).

**Figure 3:**
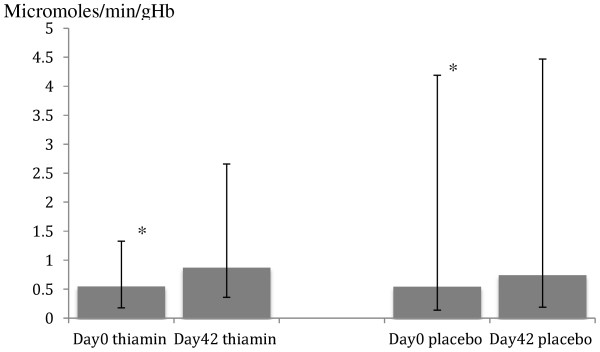
**Activated erythrocyte transketolase activity in micromoles/min/gHb, shown as median in histogram and range in error bar.** (*significant difference from D42).

**Figure 4:**
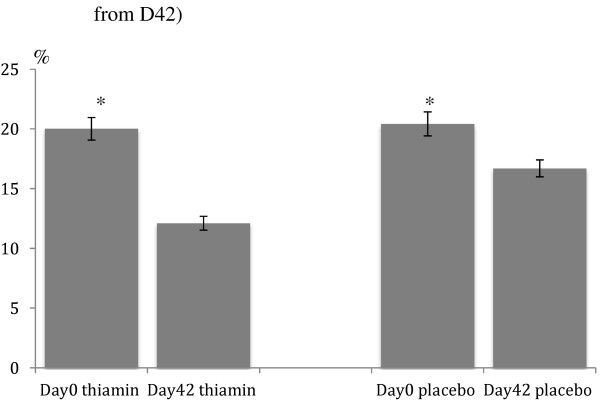
**Alpha shown as mean in histogram and 95%CI.** (*significant difference from D42).

Figures 2, 3 and 4 show the thiamin status before (day 0) and after (day 42) anti-malarial and thiamin/placebo treatments. The admission median (range) basal and activated ETK values for all study patients were 0.46 (0.12 – 3.75) and 0.54 (0.14 – 4.19) micromoles/min/gHb, respectively. These were not significantly different between thiamin and placebo groups (p = 0.98 and p = 0.78, respectively). Admission mean alpha values were also similar between the two groups (Table 4). The overall percentage of patients with thiamin deficiency (defined as alpha ≥25%) on admission was 27% and with severe thiamin deficiency (defined as alpha > 31%) 9%, with no significant differences between the groups (p = 0.13 and p = 0.33, respectively). For the 21 infants recruited (age 6–12 months), the admission median (range) basal and activated ETK were 0.52 (0.15-3.75) and 0.62 (0.21-4.19) micromoles/min/gHb, respectively. The mean alpha and the frequency of thiamin deficiency on admission were similar between the infants and children aged 1–15 years (p > 0.05).

**Table 4:** **Biochemical assays for thiamin status before and after anti-malarial treatment among patients included in the study on thiamin treatment and *Plasmodium falciparum *** **malaria in Laos**^**§**^

**Variables**	**Treatment groups**			
	**All (N = 630)**	**Thiamin (n = 314)**	**Placebo (n = 316)**	***P*-value**
Basal erythrocyte transketolase activity, median (range) micromoles/min/gHb on admission	0.46 (0.12 – 3.75)	0.46 (0.15 – 1.17)	0.45 (0.12 – 3.75)	0.98
Basal erythrocyte transketolase activity, median (range) micromoles/min/gHb at day 42*	0.70 (0.16 – 3.91)	0.78 (0.33 – 2.64)	0.64 (0.16 – 3.91)	<0.001
Activated erythrocyte transketolase activity, median (range) micromoles/min/gHb on admission	0.54 (0.14 – 4.19)	0.55 (0.18 – 1.33)	0.54 (0.14 – 4.19)	0.78
Activated erythrocyte transketolase activity, median (range) micromoles/min/gHb at day 42*	0.80 (0.19 – 4.47)	0.87 (0.36 – 2.66)	0.74 (0.19 – 4.47)	<0.001
Alpha, mean (95%CI) on admission	20 (19 – 21)	20 (19 – 21)	20 (19 – 21)	0.54
Alpha, mean (95%CI) at day 42*	14 (13 – 15)	12 (11 – 13)	17 (16 – 17)	<0.001
Alpha > 31% on admission, no (%)	55 (9)	24 (8)	31 (10)	0.33
Alpha > 31% at day 42*, no (%)	10 (2)	2 (1)	8 (3)	0.05
Alpha < 15% on admission, no (%)	185 (29)	87 (28)	98 (31)	0.36
Alpha < 15% at day 42*, no (%)	384 (63)	243 (79)	141 (46)	<0.001
Alpha = 15 – 24% on admission, no (%)	248 (39)	139 (44)	109 (34)	0.012
Alpha = 15 – 24% at day 42*, no (%)	182 (30)	56 (18)	126 (41)	<0.001
Alpha ≥ 25% on admission, no (%)	169 (27)	76 (24)	93 (29)	0.13
Alpha ≥ 25% at day 42*, no (%)	38 (6)	5 (2)	33 (11)	<0.001

^§^Data were available from 307 and 307 patients in thiamin and placebo groups, respectively.

*All values were significantly different between admission and day 42 data (*P* < 0.001) except alpha values in placebo group (*P* = 0.09).

At day 42, the overall median (range) basal and activated ETK values were 0.70 (0.16–3.91) and 0.80 (0.19–4.47) micromoles/min/gHb, respectively and these values were significantly higher in the thiamin compared to the placebo groups [0.78 (0.33–2.64) vs 0.64 (0.16–3.91), p < 0.001; and 0.87 (0.36–2.66) vs 0.74 (0.19–4.47), p < 0.001), respectively]. The mean (95%CI) day 42 alpha values were significantly lower in the thiamin [12 (11–13)%] compared to the placebo [17 (16–17)%] groups (p < 0.001). The overall proportion of patients with thiamin deficiency at day 42 was 6% and with severe thiamin deficiency was 2% and was significantly lower in the thiamin (2% overall and 1% severe deficiency) compared to the placebo (11% overall and 3% severe deficiency) groups (p < 0.001 and p = 0.05), respectively.

Following anti-malarial and thiamin/placebo treatments, there was a significant decrement in alpha. For 614 patients with paired admission and day 42 samples, the overall mean (95%CI; range) percentage change in alpha was -5.7 (-6.4 to -5.0; -53 to 20)% (paired *t*-test p < 0.001); -7.8 (-8.9 to -6.8; -53 to 20)% in the thiamin group and -3.6 (-4.6 to -2.7; -38 to 16)% in the placebo group (paired *t*-test p < 0.001 for both). The mean (95%CI) changes in basal and activated ETK (micromoles/min/gHb) values between the thiamin *versus* placebo groups were [0.34 (0.32 – 0.37) vs 0.18 (0.16 – 0.20), p < 0.001] and [0.35 (0.32 – 0.37) *vs* 0.20 (0.18 – 0.22), p < 0.001], respectively.

Higher admission and day 42 basal and activated ETK but lower admission and day 42 alpha values were observed among children compared to adults (p < 0.001 for all). The proportion of patients with thiamin deficiency (alpha ≥ 25%) was also significantly higher in the adults [89/216 (41%)] compared to children [80/414 (19%)] on admission (p < 0001) and [24/209 (11%)] vs [14/405 (3%)] at day 42 (p < 0.001). The proportion of patients with thiamin deficiency (alpha ≥ 25%) on admission and at day 42 was significantly higher in males than in females [103/317 (32%) *vs* 66/313 (21%), p = 0.001] and [27/308 (9%) *vs* 11/306 (4%), p = 0.008)], respectively. Mean alpha values and the proportion of the patients with thiamin deficiency on admission and at day 42 were not statistically different between pregnant and non-pregnant women (p > 0.05). The median (range) basal and activated ETK on admission and at day 42 were significantly higher in severe than in uncomplicated malaria patients (p < 0.01). The proportion of patients with thiamin deficiency on admission (alpha ≥ 25%) was significantly lower in severe [3/34 (9%)] compared to uncomplicated [166/596 (28%)] malaria (p = 0.01) but were similar for severe [1/30 (3%)] and uncomplicated [37/584 (6%)] groups at 42-day follow-up (p = 1.00).

### Glucose and lactate before and after anti-malarial and thiamin/placebo treatments

The mean (95%CI) whole blood glucose and lactate on admission was similar between thiamin and placebo groups (Table 1). Following anti-malarial and thiamin/placebo treatments, the mean (95%CI) glucose and lactate decreased in both thiamin and placebo groups. The overall mean (95%CI) glucose (mmol/L) and lactate (mmol/L) was significantly higher on admission than that at day 42 [6.1 (6.0 – 6.2) and 3.2 (3.1 – 3.3) *vs* 5.2 (5.1 – 5.3) and 2.6 (2.5 – 2.7), p < 0.001 for both]. The mean glucose at day 42 was not different between the groups but the mean (95%CI) lactate (mmol/L) at day 42 was slightly lower in the thiamin [2.5 (2.4 – 2.6)] than in the placebo [2.7 (2.6 – 2.8)] groups, (p = 0.048).

Among patients with severe malaria (n = 34), the mean glucose and lactate also significantly decreased following anti-malarial and thiamin/placebo treatments with the mean (95%CI) difference between admission and day 42 of 1.2 (0.6 – 1.9) mmol/L for glucose and 2.4 (1.8 – 3.0) mmol/L for lactate. The admission *versus* day 42 mean (95%CI) glucose (mmol/L) and lactate (mmol/L) among this group of patients was [6.3 (5.7 – 6.8) *vs* 5.0 (4.7 – 5.2); p < 0.001] and [5.4 (4.6 – 6.1) *vs* 2.7 (2.3 – 3.0); p < 0.001], respectively.

No consistent relationships were observed between lactate and basal and activated ETK or alpha at admission and day 42.

### Post-treatment possible adverse events

No patient had obvious symptoms or signs suggesting thiamin deficiency on admission or during or after treatment. Adverse event symptoms were recorded for only those 461 (73%) patients aged >4 years old and able to answer questions (Table 4). There were no deaths nor serious adverse events reported, including organ failure, during follow up. Except for diarrhoea, which was significantly more common in the placebo (7%) compared to thiamin (3%) groups (p = 0.04), adverse events did not significantly differ between thiamin and placebo groups. Except for the proportion of dizziness at day 1, which was significantly higher in the placebo (33%) compared to thiamin (25%) groups (p = 0.045), the proportions of the patients with dizziness, nightmare and headache following treatment at day 0, 2, 3, 7, 14, 21, 28, 35 and 42 were similar between the groups (p > 0.05). The median (range) dizziness and headache VAS scores after treatment at all time points did not differ significantly between the two groups for all patients and for only adults (p > 0.05; Table 5). The median (range) basal and activated ETK and the mean (95%CI) alpha on admission and at day 42 were not significantly different between patients with and without dizziness, headache or nightmares (p > 0.05).

**Table 5:** **Possible adverse events (AE) found in patients included in the study on thiamin treatment and *Plasmodium falciparum *** malaria in Laos*

**Variables**	**Treatment groups**			
	**All (N = 630)**	**Thiamin (n = 314)**	**Placebo (n = 316)**	***P*-value**
At least one adverse event	226/630 (36%)	101/314 (32%)	125/316 (40%)	0.053
Headache	33/438 (7.5%)	18/219 (8%)	15/219 (7%)	0.58
Insomnia	36/629 (6%)	15/313 (5%)	21/316 (7%)	0.32
Weakness	33/629 (5%)	15/313 (5%)	18/316 (6%)	0.61
Anorexia	35/629 (6%)	15/313 (5%)	20/316 (6%)	0.40
Diarrhoea	31/630 (5%)	10/314 (3%)	21/316 (7%)	0.04
Nightmare	17/439 (4%)	5/219 (2%)	12/220 (5%)	0.08
Abdominal pain	12/447 (3%)	6/221 (3%)	6/226 (3%)	0.97
Dizziness	12/438 (3%)	4/219 (2%)	8/219 (4%)	0.24
Vomiting	14/629 (2%)	4/313 (1%)	10/316 (3%)	0.10
Nausea	5/440 (1%)	2/221 (1%)	3/219 (1%)	0.65
Palpitation	5/439 (1%)	1/220 (0.5%)	4/219 (2%)	0.17
Itch	5/629 (1%)	2/313 (1%)	3/316 (1%)	0.66
Vertigo	1/438 (0.2%)	0	1/219 (0.5%)	0.32
Tinnitus	1/440 (0.2%)	0	1/220 (0.45%)	0.32
Rash	1/629 (0.2%)	1/313 (0.3%)	0	0.31
Urticaria	1/629 (0.2%)	0	1/316 (0.3%)	0.32
Irritability	0	0	0	-
Dyspnoea	1/629 (0.2%)	1/313 (0.3%)	0	0.31
Hearing loss	0	0	0	-
Paraesthesia	2/441 (0.5%)	1/221 (0.5%)	1/220 (0.5%)	0.99

*Symptoms are given for only those 461 patients aged > 4 years old and able to answer questions about these symptoms, except that parents/guardians were asked about their charges insomnia, weakness, anorexia and itch.

For both groups combined, the admission and day 42 median (range) basal and activated ETK were significantly higher in patients without any subsequent adverse events compared to those with at least one subsequent adverse effect (p < 0.0001 for all). Admission mean (95%CI) alpha was significantly lower in patients without subsequent adverse events [18% (17-19%) than in the patients with least one adverse event [22% (21-24%)] (p < 0.001). The corresponding values at day 42 were 13% (12-14%) for those without adverse events and 16% (15-17%) for those with at least one adverse event (p < 0.001). The proportion of patients with thiamin deficiency (alpha ≥ 25%) was significantly higher in those with at least one adverse event compared to those without adverse events [88/226 (39%) vs 81/404 (20%), p < 0.001] on admission and [29/218 (13%) vs 9/396 (2%), p < 0.001] at day 42, respectively. The proportion of the patients with at least one possible adverse event and the frequency of various side effects among the patients with severe malaria (n = 34) were not different between the thiamin and placebo groups (p > 0.05). Adverse events only in the first and last week were similar between thiamin and placebo groups (p > 0.05 for all).

## Discussion

A large randomized, double-blind, parallel group, placebo-controlled trial to compare the incidence of adverse events between malaria patients who were supplemented with and without thiamin in addition to standard ACT treatment was conducted. No major differences were identified between the two groups so the trial was discontinued, following the interim analyses of data from 630 recruited patients. The incidence of adverse effects, including dizziness, headache and nightmare, and the proportion of the patients with one or more subsequent adverse effects was not statistically different between the thiamin and placebo groups, suggesting that oral thiamin supplementation does not reduce adverse effects during and after anti-malarial treatment. In contrast with the post-hoc analysis of data published by Mayxay *et al.*[6], admission and day 42 median basal and activated ETK and alpha were not significantly associated with the occurrence of dizziness, headache and nightmares. However, for all patients combined, lower admission and day 42 median basal and activated ETK and higher alpha were significantly associated with the occurrence of at least one adverse event, suggesting that there be some minor relationship between thiamin status and adverse events.

Limitations of the study include a delay of 7–17 months between sample collection and ETK assays, the lack of a parenteral thiamin versus placebo arm for immediate treatment of those with severe malaria and the relative rarity of patients with severe malaria recruited to the study.

In the study of Mayxay *et al*. [6], it was unclear whether the improvement in thiamin status was a consequence of recovery from malaria or thiamin supplementation (as all patients were supplemented with thiamin 1 mg/day) or both. In this trial, the frequency and severity of thiamin deficiency were significantly lower at 42-day follow-up in both thiamin and placebo groups compared with admission with acute malaria, but with a significantly greater improvement in the thiamin treated group, suggesting that thiamin status improvement was a consequence of both recovery from malaria and supplementation. Since increment of body temperature by 1°C increases basal metabolic rate by 10%, malaria fever and increased metabolic rate augments the utilization of thiamin and, therefore, may precipitate deficiency in those with low dietary intakes [10]. Earlier research from western Thailand, that adults with severe falciparum malaria had higher alpha values than those with uncomplicated malaria, was not confirmed [5]. Indeed, in Laos it was found that the proportion of patients with raised alpha was significantly lower in patients with severe than those with uncomplicated malaria. Although the proportion of patients with severe malaria was lower in Laos, the authors were unable to explain this difference. In the current report, 27% of patients presenting with malaria had biochemical thiamin deficiency and 9% had severe deficiency on admission – similar to that reported in Mayxay *et al*. [6] at 30% and 12%, respectively, conducted at Phalanxay Clinic in the same province. In Vientiane, clinically unapparent thiamin deficiency among infants is also common: ~13% of sick infants but without clinical evidence of beriberi admitted to hospital had biochemical thiamin deficiency [7]. Severe biochemical thiamin deficiency in Lao infants with malaria could be expected, but malaria is relatively rare in Lao infants – in 2010 of 1,097 patients with malaria diagnosed in the three southern provinces of Laos, 21 (~2%) were infants (Mayxay M, personal communication).

Lao adults have multiple risk factors for thiamin deficiency including hard physical labour of rice farming, alcohol intake, consumption of polished rice and thiaminase-containing food, such as “paa dek” (fermented fish paste), and thiamin antagonists such as betel nut. The significantly higher prevalence of biochemical thiamin deficiency on admission and at 42-day follow-up among adults (≥15 years) compared to children aged <15 years old suggests that hard physical labour of the rural rice-farming adults may play an important role in biochemical thiamin deficiency [10].

## Conclusion

Thiamin supplementation did not reduce the frequency of adverse events after anti-malarial therapy among patients with falciparum malaria in southern Laos. Thiamin supplementation could help to reduce biochemical thiamin deficiency among malaria patients following anti-malarial drug treatment, but this did not have a measurable clinical or parasitological impact.

## Competing interests

The authors declare that they have no competing interests.

## Authors’ contributions

MM designed the study, recruited and followed up the patients, analysed data and drafted paper. MK and OS designed the study, recruited and followed up the patients, and revised the paper. MI designed the study, performed the molecular genetic studies, and revised the paper. TP, BH, VV, SP and NJW designed the study and revised the paper. LC oversaw the measurement of ETK activity and haemoglobin in the lysed erythrocytes. PNN designed the study, analysed data, drafted and revised the paper. All authors read and approved the final manuscript.
